# Circular noncoding RNA hsa_circ_0005986 as a prognostic biomarker for hepatocellular carcinoma

**DOI:** 10.1038/s41598-021-94074-y

**Published:** 2021-07-22

**Authors:** Gyeonghwa Kim, Ja Ryung Han, Soo Young Park, Won Young Tak, Young-Oh Kweon, Yu Rim Lee, Young Seok Han, Jung Gil Park, Min Kyu Kang, Hye Won Lee, Won Kee Lee, Deokhoon Kim, Se Young Jang, Keun Hur

**Affiliations:** 1 Department of Biochemistry and Cell Biology, School of Medicine, Kyungpook National University, 680 Gukchaebosang-ro, Jung-gu, Daegu, 41944 Republic of Korea; 2 Department of Surgery, School of Medicine, Kyungpook National University Hospital, Kyungpook National University, 130 Dongdeok-ro, Jung-gu, Daegu, 41944 Republic of Korea; 3 Department of Internal Medicine, School of Medicine, Kyungpook National University Hospital, Kyungpook National University, 130 Dongdeok-ro, Jung-gu, Daegu, 41944 Republic of Korea; 4 Department of Internal Medicine, College of Medicine, Yeungnam University, 170 Hyonchung-ro, Nam-gu, Daegu, 42415 Republic of Korea; 5 Department of Pathology, Keimyung University School of Medicine, 1035 Dalgubeol-daero, Dalseo-gu, Daegu, 42601 Republic of Korea; 6 Biostatistics, Medical Research Collaboration Center in Kyungpook National University Hospital, School of Medicine, Kyungpook National University, 135 Dongdeok-ro, Jung-gu, Daegu, 41940 Republic of Korea; 7 Department of Pathology, Asan Medical Center, University of Ulsan College of Medicine, Olympic-ro 43 gil 88, Song Pa-gu, Seoul, 05505 Republic of Korea

**Keywords:** Cancer, Molecular biology, Biomarkers, Molecular medicine

## Abstract

Circular RNAs (circRNAs) represent potential biomarkers because of their highly stable structure and robust expression pattern in clinical samples. The aim of this study was to evaluate the expression of a recently identified circRNA, hsa_circ_0005986; determine its clinical significance; and evaluate its potential as a biomarker of hepatocellular carcinoma (HCC). We evaluated hsa_circ_0005986 expression in 123 HCC tissue samples, its clinical significance, and its association with patients’ clinicopathological characteristics and survival. Hsa_circ_0005986 expression was downregulated in HCC tissues. Low hsa_circ_0005986 expression was more common in tumors larger than 5 cm [odds ratio (OR), 3.19; 95% confidence interval (CI), 1.51–6.76; *p* = 0.002], advanced TNM stage (III/IV; OR, 2.39; 95% CI, 1.16–4.95; *p* = 0.018), and higher BCLC stage (B/C; OR, 2.71; 95% CI, 1.30–5.65; *p* = 0.007). High hsa_circ_0005986 expression was associated with improved survival and was an independent prognostic factor for overall [hazard ratio (HR), 0.572; 95% CI, 0.339–0.966; *p* = 0.037] and progression-free (HR, 0.573; 95% CI, 0.362–0.906; *p* = 0.017) survival. Moreover, the circRNA–miRNA–mRNA network was constructed using RNA-seq/miRNA-seq data and clinical information from TCGA-LIHC dataset. Our findings indicate a promising role for hsa_circ_0005986 as a prognostic biomarker in patients with HCC.

## Introduction

Cancer remains a public health concern worldwide. It represents a major economic and social burden as well as a significant cause of mortality^1^. In 2012, there were an estimated 14 million and 8 million new cancer cases and cancer-related deaths, respectively. This increased to more than 18 million and 9 million, respectively, in 2018, which is evidence of the rapidly increasing rates of cancer incidence and mortality^2,3^. In particular, liver cancer is a commonly diagnosed cancer worldwide and is the fourth leading cause of cancer-related mortality. Hepatocellular carcinoma (HCC) accounts for approximately 80% of primary liver cancers^2^ and is associated with poor patient prognosis. HCC originates primarily in chronically damaged liver (i.e., liver cirrhosis resulting from chronic viral hepatitis B or C or long-term alcohol consumption). Frequent recurrence of HCC limits treatment options because of the underlying liver disease and impaired liver function.

Because of the poor prognosis associated with HCC, the identification of biomarkers is essential for predicting patient prognosis and survival and tumor recurrence as well as for determining suitable treatment options. The recent development of high-throughput sequencing techniques and advances in bioinformatics has resulted in an increase in the number of candidate biomarkers^4^. Noncoding RNAs such as long noncoding RNAs, microRNAs (miRNAs), and circular RNAs (circRNAs) are some potential biomarkers that may have relevance to HCC.

Circular RNAs (circRNAs) are a class of highly stable, single-stranded RNAs that form a loop through covalent binding. They are synthesized either from coding or noncoding genomic regions. Whereas circRNAs are formally known to be noncoding, recent evidence indicates the existence of protein-coding circRNAs^5^. In contrast to linear RNAs, circRNAs are formed through a back-splicing event, which occurs via the linkage of downstream 3′ and upstream 5′ splice sites to form covalent and canonical bonds^6^. Exons, introns, or both may serve as substrates for circRNA back-splicing. This produces four types of circRNAs: exonic (EcircRNAs), circular intronic (ciRNAs), exon–intron (EIciRNAs), and tRNA intronic (tricRNAs)^7–9^. Most circRNAs are EcircRNAs. ciRNAs are localized abundantly in the nucleus and show minute enrichment for target miRNA sites. Importantly, the fact that ciRNA knockdown can lead to the downregulation of the expression of its corresponding parental gene suggests that ciRNAs are involved in positively modulating transcription catalyzed by RNA polymerase II^9^. EIciRNAs are RNA molecules in which the exons are separated by retained introns. The nuclear abundance of both ciRNAs and EIciRNAs suggests that they are involved in transcriptional and post-transcriptional events^7,8,10,11^. Pre-tRNA splicing into two parts by specific enzymes gives rise to tRNA and tricRNA—a unique class of ciRNA^12^.

CircRNAs are present predominantly in the cytoplasm. They contain miRNA response elements (MREs) and serve as sponges for miRNAs, thereby downregulating their expression. This results in decreased miRNA-mediated mRNA degradation or translational repression.

Although the exact function of circRNAs remains unclear, many studies have revealed their involvement in both physiological and pathological processes^13^, including cell aging^14^, tissue development^15,16^, and neurological disorders such as Alzheimer’s disease^17^. Furthermore, circRNAs are expressed in various cancers, including glioblastoma multiforme^18^, colorectal^19,20^, breast^21,22^, gastric^23^, and bladder^24^ cancers as well as HCC^25,26^. circRNAs may serve as miRNA sponges^27^ and may be involved in epithelial–mesenchymal transition (EMT)^28^ and development^29^ of various cancers. Collectively, these findings indicate that circRNAs play important roles in various cellular processes and may serve as clinical biomarkers.

The aim of this study was to evaluate the expression of a recently identified circRNA, hsa_circ_0005986, determine its clinical significance, and evaluate its potential as a biomarker for HCC.

## Materials and methods

### Patients and tissue samples

**Figure 1:**
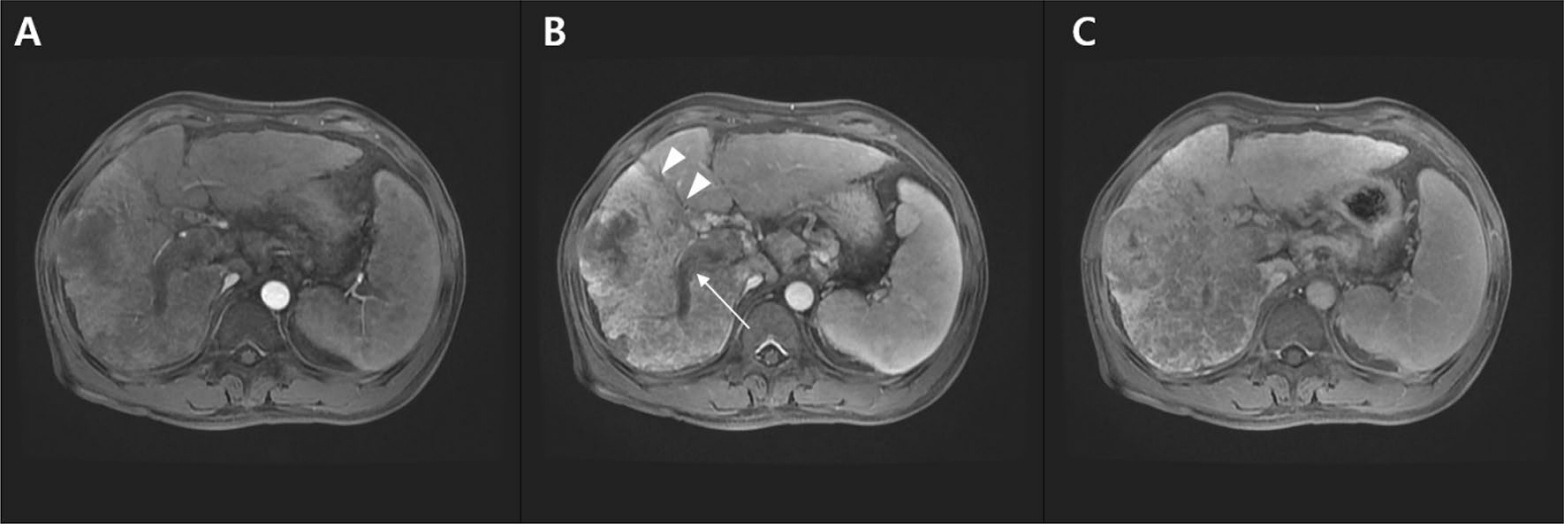
MRI image of a patient with cancer. Gadoxetic acid disodium-enhanced liver MRI images of a patient with HCC. (**A**) A huge mass replacing the right lobe of the liver in the arterial phase. (**B**) The mass (arrowhead) and portal vein thrombosis (arrow) are more prominent in the portal phase. (**C**) The mass shows washout in the venous phase.

This study included 162 patients with HCC (Fig. 1) who underwent diagnostic biopsy or surgical resection at Kyungpook National University Hospital, Republic of Korea, between March 2015 and August 2016. Thirty patients who had been previously treated for HCC and nine patients who were lost to follow-up were excluded from the study, resulting in a final sample size of 123 evaluable patients. Tissue samples were obtained by liver biopsy or surgical resection. Liver biopsy was performed to confirm HCC diagnosis and to rule out the presence of other tumors. Patients underwent surgical resection (n = 19) or radiofrequency ablation (n = 47) as curative treatment (n = 66, 53.7%) or transarterial chemoembolization (n = 9), sorafenib (n = 12) or best supportive care (*n* = 36) as non-curative treatment. This study was conducted according to local ethical guidelines, in accordance with the Declaration of Helsinki.

For post-treatment monitoring, imaging was conducted every 3–6 months using contrast-enhanced dynamic computed tomography (CT) or gadoxetic acid disodium-enhanced liver magnetic resonance imaging (MRI). We defined overall survival as the time between the date of initial HCC diagnosis and either the date of death from any cause or the date of last contact with the patient during follow-up examination. Progression-free survival was defined as the time between the initial date of HCC diagnosis and either the first event of recurrence or progression or until death from any cause. The recurrence of HCC was recognized if a tumor exceeded 1 cm and showed characteristic CT or MRI contrast enhancement in the arterial phase and washout in the venous or delayed phase. Response Evaluation Criteria in Solid Tumors (version 1.1) was used to evaluate tumor response. HCC specimens and adjacent non-tumor tissue specimens were immediately stored at 4 °C for 24 h in RNAlater reagent (Ambion; Life Technologies, Carlsbad, CA, USA) and then stored at − 80 °C. We recorded the patients’ age and sex, number and size of tumor, presence of macrovascular invasion, tumor node metastasis (TNM) stage, Barcelona Clinic Liver Cancer (BCLC) stage, Child–Turcotte–Pugh (CTP) category of liver function, alpha-fetoprotein (AFP) level, and other pertinent laboratory data. Cancer staging was performed according to the criteria of the American Joint Committee on Cancer (8th edition) and also following BCLC staging criteria. The ethical committee of our institution (Kyungpook National University Hospital) approved the study (#KNUH-2014-04-056-001), and all patients provided written informed consent prior to sample collection.

### Total RNAs extraction and cDNA synthesis

QIAzol Lysis Reagent (Qiagen, Hilden, Germany) was used to extract total RNAs from the frozen specimens according to the manufacturer’s instructions. We used a NanoDrop 2000 spectrophotometer (Thermo Scientific, Waltham, MA, USA) to determine RNA concentration as well as its purity. A High-Capacity cDNA Reverse Transcription kit (Applied Biosystems, Foster City, CA, USA) was used to reverse transcribe cDNA according to the manufacturer’s instructions.

### Quantitative real-time polymerase chain reaction (qRT-PCR)

We used SYBR Green PCR Master Mix (Applied Biosystems) to perform qRT-PCR. The expression of hsa_circ_0005986 was normalized to that of glyceraldehyde 3-phosphate dehydrogenase (GAPDH) and then quantified using the 2^−ΔΔCt^ method. All primers were synthesized by Bionics (Seoul, Korea). In order to amplify only hsa_circ_0005986, but not linear form of RNA, the primers were designed by considering the backsplice junctions of circRNA (Supplementary Fig. 1). The following primer sequences were used: 5′-GAA ACT GGC TGC GAT ATG TG-3′ (forward) and 5′-CAC AGG CTC AGT AGT GTT CTT TAA A-3′ (reverse) for hsa_circ_0005986 and 5′-GGA AGG TGA AGG TCG GAG TC-3′ (forward) and 5'-GTT GAG GTC AAT GAA GGG GTC-3' (reverse) for GAPDH. qRT-PCR was performed in triplicate, and specific target amplification was confirmed by melting curve analysis.

### Statistical analysis

For descriptive statistics, categorical data were expressed as number (%) and numerical data as the mean and standard deviation for normally distributed data and as the median with interquartile range for non-normally distributed data. A paired t-test was used to analyse differences in hsa_circ_0005986 expression between HCC and adjacent non-tumor tissue. We used the chi-square or Fisher’s exact probability test to compare clinicopathological characteristics between two groups with different hsa_circ_0005986 expressions. The Kaplan–Meier method was used to generate survival curves, and the log-rank test was conducted to compare survival curves between groups. The prognostic performances^30^ of hsa_circ_0005986 were expressed as specificity, sensitivity, and area under the receiver operating curve (AUC). To determine the predictors of survivals, univariate and multivariate analyses based on a Cox proportional hazards model were performed. *p*-values of < 0.05 were considered statistically significant. Values that were statistically significant in univariate analyses were included in multivariate analyses, with a *p*-value of < 0.1. We conducted all the analyses using SAS version 9.4 software (SAS Institute Inc., Cary, NC, USA), and GraphPad Prism 6 program for Windows (GraphPad Software, La Jolla, CA, USA) was used to generate figures.

### Construction of the circRNA–miRNA–mRNA network^31,32^

Publicly available sequencing data (RNA-seq, miRNA-seq and clinical information data) related to HCC were obtained from The Cancer Genome Atlas (TCGA) using gdc-rnaseq-tool (https://github.com/cpreid2/gdc-rnaseq-tool). Differentially expressed genes and miRNA (|log2-fold change|≥ 1 and adjust *p* value < 0.05) were analyzed using the DEseq2^33^ R package (version 1.30.1) for further analysis. A co-expression network was constructed using a WGCNA package^34^ (version 1.70-3) in the R software (version 4.0.3). Potential target miRNAs of hsa_circ_0005986 were predicted via circBank^35^. The overlapping part of the target miRNAs from co-expressed miRNAs from WGCNA were selected. The potential target genes of the selected miRNA were predicted using mirDB^36^. The overlapping part of the target genes from co-expressed genes from WGCNA were selected. The circRNA–miRNA–mRNA network was visualized using Cytoscape (version 3.8.2 for Mac).

### Ethics approval and consent to participate

The ethical committee of our institution (Kyungpook National University Hospital) approved the study (#KNUH-2014-04-056-001), and all patients provided written informed consent prior to sample collection.

### Consent for publication

Informed consent for publication was obtained from all participants.

## Results

### Baseline characteristics of patients with HCC

Table 1 shows the baseline characteristics of patients with HCC. The patients predominantly comprised males (85.4%), and the mean age was 60.3 years. Liver disease was primarily caused by hepatitis B (61.0%), alcohol consumption (25.2%), hepatitis C (8.9%), or hepatitis B and C virus co-infection (1.6%). Sixty-five (52.8%) patients presented with a single tumor and 58 (47.2%) had multiple tumors. The tumor size was > 5 cm in 66 (53.7%) patients and ≤ 5 cm in 57 (46.3%) patients. Vessel invasion was observed in 44 (35.8%) patients. Overall, 66 (53.7%) patients had TNM stage I or II tumor and 57 (46.4%) had TNM stage III or IV tumor. Moreover, 59 (48.0%) patients had cancer at BCLC stages 0 and A and 64 (52.0%) patients had cancer at BCLC stages B and C. The CTP class was A in 105 (85.4%) patients and B in 18 (14.6%) patients. The median aspartate transaminase, alanine transaminase, bilirubin, albumin, and AFP levels and prothrombin time were 52.0 U/L, 37.0 U/L, 0.8 mg/dL, 3.9 g/dL, 52.8 ng/mL, and 12.6 s, respectively. Of these clinical characteristics, sex and etiology of liver disease were not statistically different between the curative treatment and non-curative treatment groups.

**Table 1:** Baseline characteristics of patients with HCC.

Clinical characteristics	Curative (n = 66)	Non-curative (n = 57)	Total (*n* = 123)	*p*-value
Age (years)	62.7 ± 10.8	57.5 ± 10.7	60.3 ± 11.1	0.008*
**Sex**				0.667
Male	55	50	105 (85.4%)	
Female	11	7	18 (14.6%)	
**Etiology**				0.364
HBV	42	33	75 (61.0%)	
HCV	8	3	11 (8.9%)	
Alcohol	13	18	31 (25.2%)	
HBV + HCV	0	2	2 (1.6%)	
NASH	1	0	1 (0.8%)	
Cryptogenic	2	1	3 (2.4%)	
**Tumor number**				< 0.001*
Single	54	12	66 (53.7%)	
Multiple	12	45	57 (46.3%)	
**Size of tumor (cm)**				< 0.001*
≤ 5	52	6	58 (47.2%)	
> 5	14	51	65 (52.8%)	
**Vessel invasion**				< 0.001*
No	62	18	80 (65.0%)	
Yes	4	39	43 (35.0%)	
**TNM stage**				< 0.001*
I	51	1	52 (42.3%)	
II	10	4	14 (11.4%)	
III	3	11	14 (11.4%)	
IV	2	41	43 (35.0%)	
**BCLC stage**				< 0.001*
O/A	56	4	60 (48.8%)	
B/C	10	53	63 (51.2%)	
**CTP class**				< 0.001*
A	65	41	106 (86.2%)	
B	1	16	17 (13.8%)	
AST (U/L)	36.0 [26.0–58.0]*	68.0 [47.0–120.0]*	52.0 [30.5–76.5]*	< 0.001*
ALT (U/L)	32.0 [25.0–46.0]*	42.0 [29.0–64.0]*	37.0 [26.0–55.5]*	0.018*
Bilirubin (mg/dL)	0.6 [0.5–0.9]*	1.1 [0.6–1.6]*	0.8 [0.5–1.3]*	< 0.001*
Albumin (g/dL)	4.0 ± 0.5	3.5 ± 0.6	3.8 ± 0.6	< 0.001*
Prothrombin time (s)	12.1 [11.4–12.8]*	13.0 [12.0–13.9]*	12.6 [11.9–13.4]*	< 0.001*
AFP (ng/mL)	10.6 [4.8–142.9]*	1394.0 [46.6–21,551.0]*	47.6 [7.5–2,659.5]*	< 0.001*

*HBV* hepatitis B virus; *HCV* hepatitis C virus; *NASH* nonalcoholic steatohepatitis; *TNM* tumor node metastasis; *BCLC* Barcelona Clinic Liver Cancer; *CTP* Child–Turcotte–Pugh; *AST* aspartate transaminase; *ALT* alanine transaminase; *AFP* alpha-fetoprotein; *median [25%–75% interquartile range].

### Downregulation of hsa_circ_0005986 expression in HCC tissues and advanced HCC

**Figure 2:**
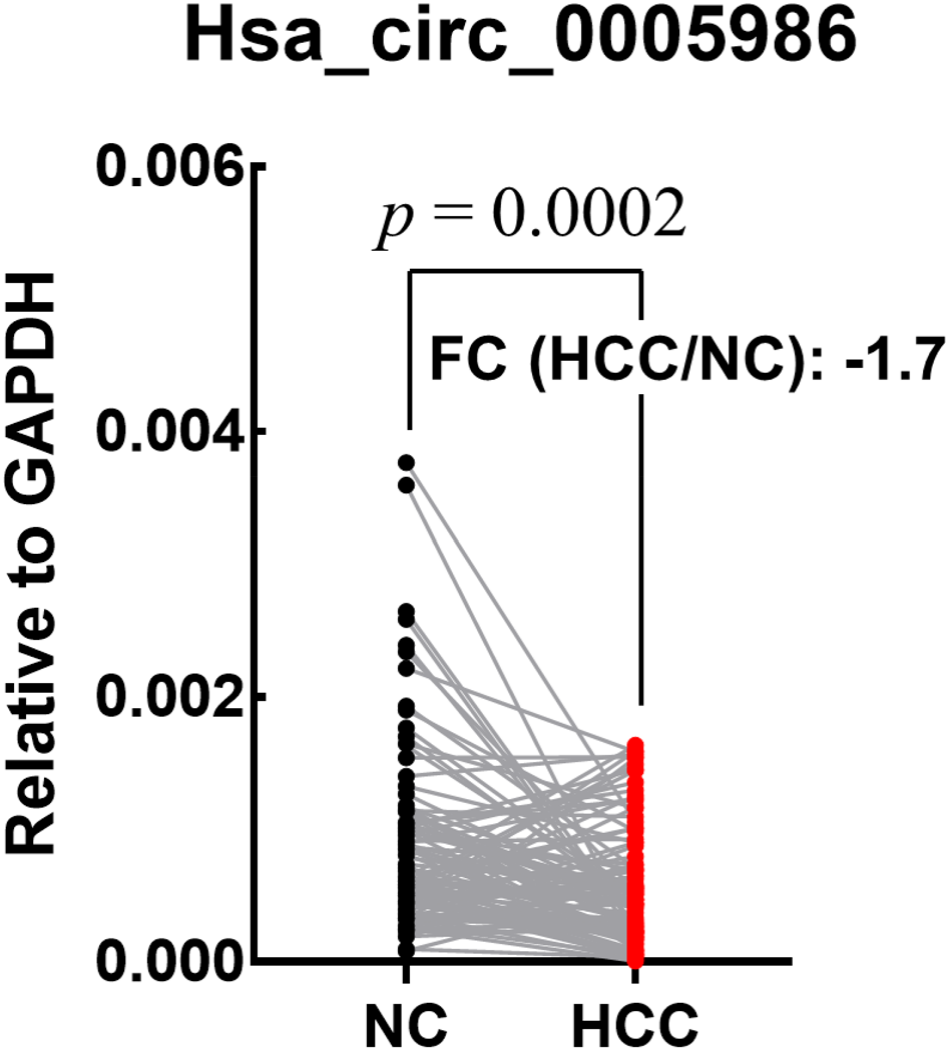
Dot-plot of hsa_circ_0005986 expression in non-cancerous (NC) and hepatocellular carcinoma (HCC) tissues. Hsa_circ_0005986 expression in HCC tissues was lower than [fold change (FC): -1.7] that in NC tissues (*p* = 0.0002).

**Figure 3:**
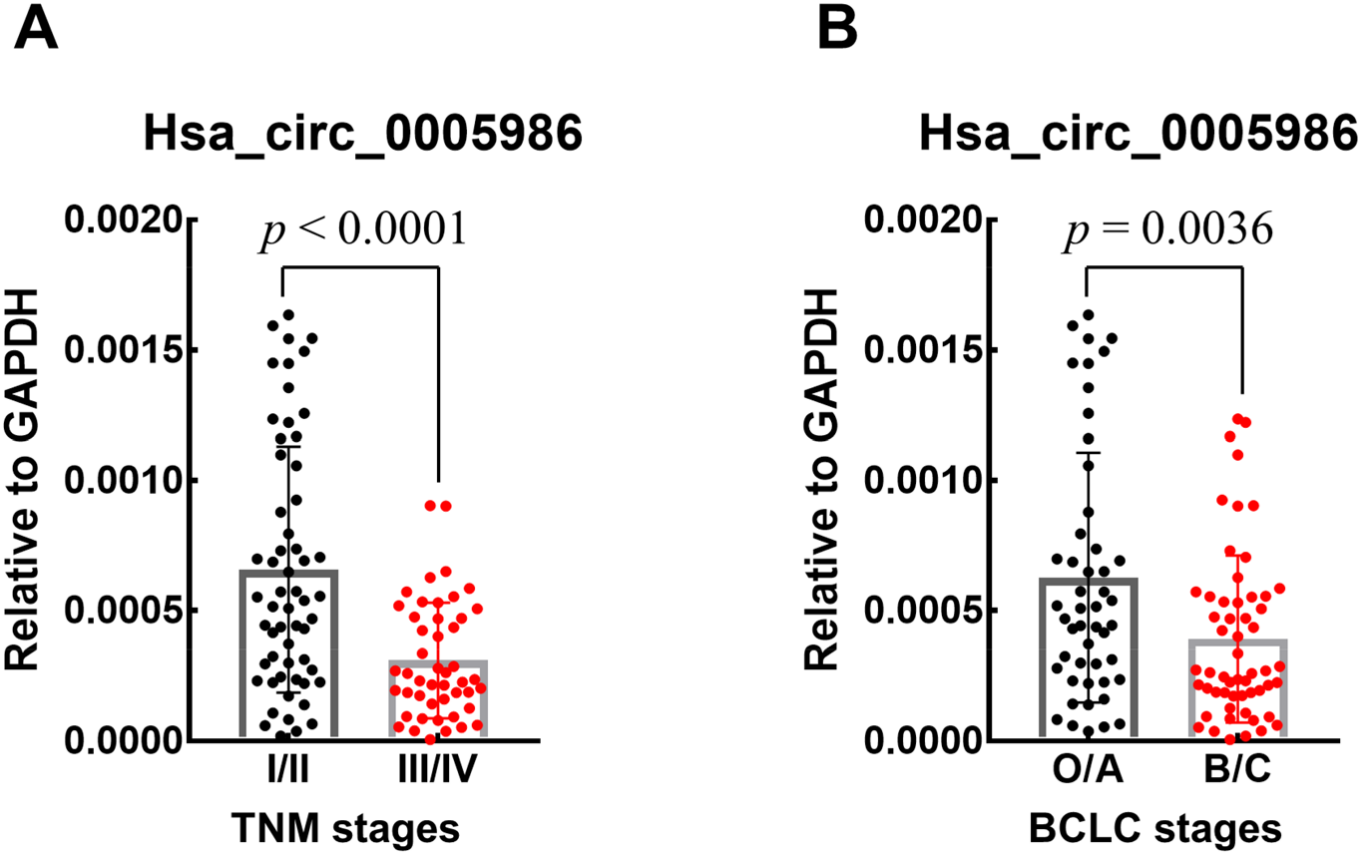
Dot-plot of hsa_circ0005986 expression according to the TNM and BCLC stage. Hsa_circ_0005986 expression was decreased in TNM stage III/IV (A) (*p* < 0.0001) and BCLC stage B/C (B) (*p* = 0.0036). *TNM* tumor-node-metastasis; *BCLC* Barcelona Clinic Liver Cancer.

We found that the expression of hsa_circ_0005986 was significantly downregulated in HCC tissues compared with that in adjacent non-tumor tissues (Fig. 2). In addition, we found that the expression of hsa_circ_0005986 in TNM stages III and IV was significantly lower than that in stages I and II. Similarly, lower hsa_circ_0005986 expression was observed in BCLC stages B and C compared with that in stages 0 and A (Fig. 3).

### Correlation between hsa_circ_0005986 expression and clinicopathological characteristics of patients with HCC

Table 2 shows the differences in patients’ characteristics based on the expression level of hsa_circ_0005986. Based on hsa_circ_0005986 expression, all patients were classified into a high-expression (≥ 0.0004347) or low-expression (< 0.0004347) group. The cutoff value was selected as the optimal value from the area under the curve (AUC) analysis of the time-dependent receiver operating characteristic (ROC) curve that maximized the sum of the specificity and sensitivity for predicting survival. Low hsa_circ_0005986 expression was associated with tumors larger than 5 cm [odds ratio (OR), 3.19; 95% confidence interval (CI), 1.51–6.76; *p* = 0.002), advanced TNM stage (III/IV; OR, 2.39; 95% CI, 1.16–4.95; *p* = 0.018), and higher BCLC stage (B/C; OR, 2.71; 95% CI, 1.30–5.65; *p* = 0.007).

**Table 2:** Correlation between hsa_circ_0005986 expression and clinical parameters in patients with HCC.

Clinical characteristics	Hsa_circ_0005986		
	Low (n = 55)	High (n = 68)	*p*-value
**Age (years)**			0.220
≤ 60	32 (58.2%)	32 (47.1%)	
> 60	23 (41.8%)	36 (52.9%)	
**Sex**			0.626
Male	46 (83.6%)	59 (86.8%)	
Female	9 (16.4%)	9 (13.2%)	
**Tumor number**			0.066
Single	24 (43.6%)	41 (60.3%)	
Multiple	31 (56.4%)	27 (39.7%)	
**Tumor size (cm)**			0.002*
≤ 5	17 (30.9%)	40 (58.8%)	
> 5	38 (69.1%)	28 (41.2%)	
**Vessel invasion**			0.102
No	31 (56.4%)	48 (70.6%)	
Yes	24 (43.6%)	20 (29.4%)	
**TNM stage**			0.018*
I/II	23 (41.8%)	43 (63.2%)	
III/IV	32 (58.2%)	25 (36.8%)	
**BCLC stage**			0.007*
O/A	19 (34.5%)	40 (58.8%)	
B/C	36 (65.5%)	28 (41.2%)	
**CTP classification**			0.626
A	46 (83.6%)	59 (86.8%)	
B	9 (16.4%)	9 (13.2%)	
**AFP (ng/mL)**			0.082
≤ 400	32 (58.2%)	49 (73.1%)	
> 400	23 (41.8%)	18 (26.9%)	
**Chronic hepatitis B**			0.842
No	22 (40.0%)	26 (38.2%)	
Yes	33 (60.0%)	42 (61.8%)	

*TNM* tumor node metastasis; *BCLC* Barcelona Clinic Liver Cancer; *CTP* Child–Turcotte–Pugh; *AFP* alpha-fetoprotein.

*p < 0.05.

### Correlation between hsa_circ_0005986 expression and survival of patients with HCC

**Figure 4:**
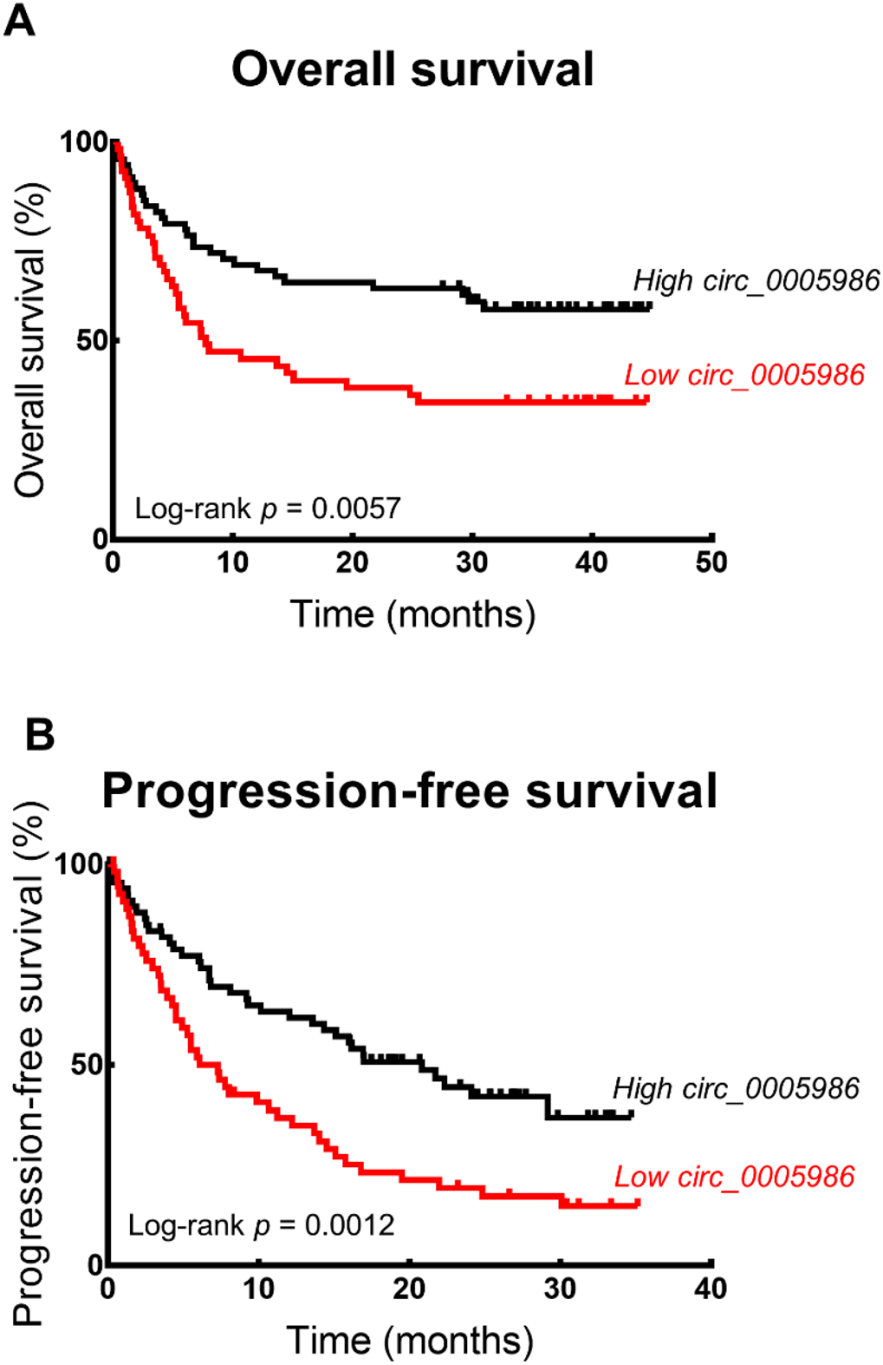
Survival curves according to hsa_circ_0005986 expression. Overall survival (**A**) (log-rank test, *p* = 0.0057) and progression-free survival (**B**) (log-rank test, *p* = 0.0012) rates were decreased in the high hsa_circ_0005986 expression group.

The overall survival of patients was significantly different according to hsa_circ_0005986 expression (Fig. 4A). The cumulative 1-, 2-, and 3-year overall survival rates were 45.5%, 38.2%, and 34.5%, respectively, in the low-expression group and 67.6%, 61.8%, and 56.6%, respectively, in the high-expression group.

The AUC of hsa_circ_0005986 for predicting survival was 0.633 (95% CI: 0.542–0.718, *p* = 0.009) with a sensitivity of 54.7% and specificity of 71.2%. The AUC of hsa_circ_0005986 for predicting progression was 0.673 (95% CI: 0.582–0.754, *p* = 0.001) with a sensitivity of 53.7% and specificity of 80.5%.

The univariate analysis of prognostic factors for overall survival in patients with HCC (Table 3) demonstrated the following significant predictors for overall survival: high hsa_circ_0005986 expression [hazard ratio (HR), 0.504; 95% CI, 0.307–0.828; *p* = 0.007], multiple tumors (HR, 3.655; 95% CI, 2.171–6.152; *p* < 0.001), large tumors (> 5 cm; HR, 10.083; 95% CI, 5.083–20.000; *p* < 0.001), vessel invasion (HR, 10.521; 95% CI, 5.983–18.502; *p* < 0.001), AFP level > 400 ng/mL (HR, 5.108; 95% CI, 3.077–8.481; *p* < 0.001), poor CTP class (HR, 3.283; 95% CI, 1.829–5.892; *p* < 0.001), and curative treatment (HR, 0.089; 95% CI, 0.048–0.167; *p* < 0.001).

Multivariate analysis identified the following independent prognostic factors for overall survival: high hsa_circ_0005986 expression (HR, 0.572; 95% CI, 0.339–0.966; *p* = 0.037), vessel invasion (HR, 3.364; 95% CI, 1.678–6.746; *p* = 0.001), AFP level > 400 ng/mL (HR, 1.846; 95% CI, 1.041–3.273; *p* = 0.036), and curative treatment (HR, 0.383; 95% CI, 0.158–0.929; *p* = 0.034).

**Table 3:** Prognostic factors for overall survival in univariate and multivariate analyses.

Factor	Univariate analysis		Multivariate analysis	
	Hazard ratio (95% CI)	*p-*value	Hazard ratio (95% CI)	*p-*value
Hsa_circ_0005986 (High expression)	0.504 (0.307–0.828)	0.007*	0.572 (0.339–0.966)	0.037*
Age (> 60 years)	0.772 (0.471–1.266)	0.305		
Sex (Female)	0.948 (0.468–1.918)	0.881		
Tumor number (Multiple)	3.655 (2.171–6.152)	< 0.001*	1.579 (0.843–2.956)	0.153
Tumor size (> 5 cm)	10.083 (5.083–20.000)	< 0.001*	2.132 (0.892–5.096)	0.089
Vessel invasion	10.521 (5.983–18.502)	< 0.001*	3.364 (1.678–6.746)	0.001*
AFP (> 400 ng/mL)	5.108 (3.077–8.481)	< 0.001*	1.846 (1.041–3.273)	0.036*
CTP classification (B vs. A)	3.283 (1.829–5.892)	< 0.001*	1.360 (0.712–2.597)	0.352
Curative treatment	0.089 (0.048–0.167)	< 0.001*	0.383 (0.158–0.929)	0.034*

*CI* confidence interval; *AFP* alpha-fetoprotein; *CTP* Child–Turcotte–Pugh.

*p < 0.05.

### Correlation between hsa_circ_0005986 expression and progression-free survival in patients with HCC

Progression-free survival differed significantly between patients depending on the hsa_circ_0005986 expression level (Fig. 4B). The cumulative 1-, 2-, and 3-year progression-free survival rates were 36.8%, 19.4%, and 14.8%, respectively, in the low-expression group and 61.8%, 43.1%, and 35.8%, respectively, in the high-expression group.

Univariate analysis of the factors associated with progression-free survival (Table 4) revealed the following significant prognostic factors: high hsa_circ_0005986 expression (HR, 0.492; 95% CI, 0.317–0.763; *p* = 0.002), multiple tumors (HR, 2.592; 95% CI, 1.666–4.032; *p* < 0.001), tumor size < 5 cm (HR, 5.239; 95% CI, 3.210–8.550; *p* < 0.001), vessel invasion (HR, 6.001; 95% CI, 3.717–9.686; *p* < 0.001), AFP level > 400 ng/mL (HR, 3.769; 95% CI, 2.407–5.903; *p* < 0.001), poor CTP class (HR, 3.257; 95% CI, 1.886–5.624; *p* < 0.001), and curative treatment (HR, 0.164; 95% CI, 0.101–0.267; *p* < 0.001).

Multivariate analysis revealed the following independent prognostic factors for progression-free survival: high hsa_circ_0005986 expression (HR, 0.573; 95% CI, 0.362–0.906; *p* = 0.017), vessel invasion (HR, 2.657; 95% CI, 1.435–4.921; *p* = 0.002), and AFP level > 400 ng/mL (HR, 1.702; 95% CI, 1.001–2.892; *p* = 0.049).

**Table 4:** Prognostic factors for progression-free survival in univariate and multivariate analyses.

Factor	Univariate analysis		Multivariate analysis	
	Hazard ratio (95% CI)	*p-*value	Hazard ratio (95% CI)	*P* value
Hsa_circ_0005986 (High expression)	0.492 (0.317–0.763)	0.002*	0.573 (0.362–0.906)	0.017*
Age (> 60 years)	0.715 (0.461–1.108)	0.133		
Sex (Female)	0.948 (0.502–1.789)	0.869		
Tumor number (Multiple)	2.592 (1.666–4.032)	< 0.001*	1.196 (0.691–2.069)	0.522
Tumor size (> 5 cm)	5.239 (3.210–8.550)	< 0.001*	1.449 (0.715–2.937)	0.303
Vessel invasion	6.001 (3.717–9.686)	< 0.001*	2.657 (1.435–4.921)	0.002*
AFP (> 400 ng/mL)	3.769 (2.407–5.903)	< 0.001*	1.702 (1.001–2.892)	0.049*
CTP classification (B vs. A)	3.257 (1.886–5.624)	< 0.001*	1.616 (0.866–3.013)	0.131
Curative treatment	0.164 (0.101–0.267)	< 0.001*	0.506 (0.239–1.073)	0.076

*CI* confidence interval; *AFP* alpha-fetoprotein; *CTP* Child–Turcotte–Pugh.

*p < 0.05.

### Prediction of hsa_circ_0005986 function

**Figure 5:**
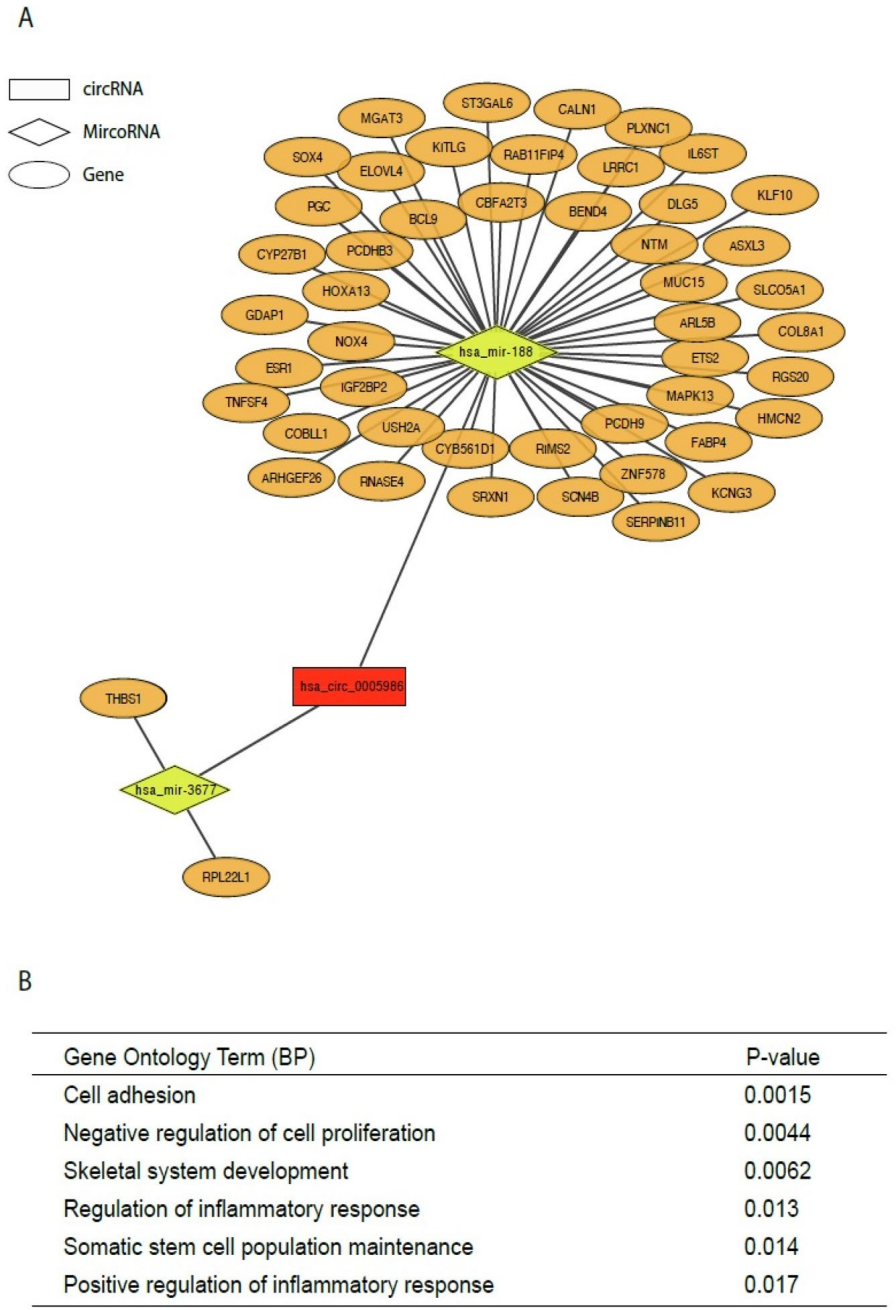
Construction of circRNA–miRNA–mRNA network. (**A**) An Illustration of circRNA-miRNA-mRNA interaction network (**B**) gene ontology analysis of target genes. *BP* biological process.

We obtained RNA-seq/miRNA-seq data and clinical information from the TCGA-LIHC dataset, including 425 RNA-seq and 425 miRNA-seq samples (50 normal samples and 374 tumors for RNA-seq; 375 tumors for miRNA-seq), and differentially expressed mRNAs and miRNAs were identified (9004 for mRNA and 310 for miRNA). To further investigate the target of hsa_circ_005986, circBank and WGCNA were performed. Two miRNAs were predicted as target of hsa_circ_005986. The target genes of hsa-mir-3677 and hsa-mir-188 were predicted using mirDB and WGCNA. Finally, we used 2 miRNAs and 52 genes to construct a circRNA–miRNA–mRNA network (Fig. 5A). Figure 5B shows the related biological processes, such as, cell adhesion (*p* = 0.0015), negative regulation of cell proliferation (*p* = 0.0044), skeletal system development (*p* = 0.0062), regulation of inflammatory response (*p* = 0.013), somatic stem cell maintenance (*p* = 0.014), and positive regulation of inflammatory response (*p* = 0.017), all of which were statistically significant.

## Discussion

The prognosis of patients with HCC is poor^37^, in part because most HCC cases are diagnosed at an advanced stage, thereby limiting the treatment options available and resulting in poor overall prognosis^38^. HCC exhibits various clinical characteristics because of the diverse etiologies associated with the underlying liver disease, impaired hepatic function, and tumor biology, even between the same disease stage. Because the same HCC stage may result in different clinical outcomes, identifying factors that affect prognosis is of great importance. In addition to clinicopathological factors, novel biomarkers can predict tumor recurrence or progression and are currently an active area of investigation.

AFP is a widely used biomarker for HCC; however, it is not specific enough for screening and diagnosing HCC^39^. Limited progress has been made in identifying new candidate markers, such as des-gamma carboxyprothrombin or fucosylated AFP, which have exhibited low accuracy during clinical evaluation^40^. Therefore, discovering novel markers is imperative to facilitate timely and early HCC diagnosis and improve treatment success and survival of patients with HCC.

CircRNAs have recently been shown to be potential biomarkers for many cancers, including HCC^41–43^. CircRNAs are promising biomarkers because of their highly stable structure and robust expression patterns in clinical samples. Owing to the covalently closed structure that prevents their cleavage and degradation by exonucleases, circRNAs are highly stable in blood^44^, saliva^45^, and exosomes^46^. As such, they show enormous potential as cancer biomarkers. A previous study showed that the median half-life of tested circRNAs was 2.5-fold longer than that of their linear counterparts from the same gene and showed that circRNAs were stably expressed, whereas mRNA and microRNA expression levels changed within minutes^47^. Recently, the potential of some circRNAs (circ_005075 and circ_0016788) as HCC diagnostic biomarkers has been suggested^48,49^. For example, circ-CDYL was upregulated in the early stages of HCC but showed a low AUC value, i.e., 0.64^50^, which was lower than those reported for circ_005075 and circ_0016788 (0.94 and 0.85, respectively)^48,49^. Similarly, the development of prognostic biomarkers for HCC has progressed, and some candidates have been identified. Upregulated circ_001569, circ_0008450, and circ_0000267 expressions were associated with poor HCC prognosis suggesting that these circRNAs are independent prognostic markers for HCC^51–53^.

By regulating cell proliferation, migration, invasion, apoptosis, metastasis, and EMT, circRNAs play either an oncogenic or a suppressive role in the progression of HCC^25,54–57^. The majority of these processes are regulated by circRNAs via miRNA sponging. For instance, to facilitate tumorigenesis, hsa_circ_101280 serves as a sponge for miR-375 and upregulates JAK2 expression, thereby promoting the proliferation of HCC cells as well as suppressing tumor cell apoptosis^54^. In addition, the oncogenic role of circSLC3A2 was shown to be dependent on the regulation of PPM1F expression by sponging miR-490-3p^56^. Another study involving HCC cells revealed higher circASAP1 expression in cells with higher metastatic potential. In addition, circASAP1 was shown to regulate the miR-326/miR-532-5p-MAPK1 pathway, thereby promoting the proliferation of tumor cells in HCC as well as metastasis^43^. Conversely, circMTO1 and hsa_circ_0001445 were found to promote the expression of the tumor suppressor genes *p21*^25^ and *TIMP3*^57^, respectively. These actions are based on the sponging of miR-9^25^, miR-17-3p, and miR-181b-5p^57^.

Our study revealed a clear relationship between the patients’ characteristics and hsa_circ_0005986 expression. We showed that hsa_circ_0005986 exhibited reduced expression in HCC and demonstrated that it was associated with clinical and pathological characteristics of patients with HCC. To our knowledge, this is the first study to validate hsa_circ_0005986 as a prognostic biomarker in a HCC cohort by performing survival and regression analyses. In 2017, Fu et al. showed that hsa_circ_0005986 sponged miR-129-5p to regulate NOTCH1 expression in HCC. Downregulated hsa_circ_0005986 expression led to the liberation of miR-129-5p, leading to lower NOTCH1 expression. This was coupled with an accelerated G0/G1 to S phase transition to promote cell proliferation^58^. The authors also showed an association between hsa_circ_0005986 expression and patient data, which is consistent with our results, except for the correlation of family history with chronic hepatitis B. We did not find an association between hsa_circ_0005986 expression and chronic hepatitis B infection. Moreover, the previous study did not include survival or progression data from 81 patients with HCC. The study by Fu et al. focused on the mechanistic aspect of hsa_circ_0005986 to be considered as a biomarker. On the contrary, our study focused more on the validation of hsa_circ_0005986 as a prognostic biomarker, along with other clinical predictors, based on survival and progression data from a larger number of patients with HCC. Therefore, the difference between this study and the previous study is the validation of a potential prognostic biomarker, hsa_circ_0005986, in a larger HCC cohort, which might be the originality of our study.

We analyzed the whole genome mRNA–miRNA–hsa_circ_0005986 and its interaction network for predicting the role of hsa_circ_0005986 in HCC. On gene ontology analysis, cell adhesion, inhibition of cell proliferation, and skeletal system development were all possibly related to the migration, proliferation, and invasion of HCC. In our study, hsa_circ_0005986 is downregulated in HCC compared with the background liver and also in the higher stages of HCC. These findings were compatible with the potential function of hsa_circ_0005986 as an inhibitor of proliferation. Regulation of the inflammatory response, promotion of the inflammatory response, and somatic stem cell population maintenance might be related to HCC development. Moreover, for predicting the potential mechanism of hsa_circ_0005986, RNA modification or a microbiome should be discussed. Aside from circRNAs relevant to HCC progression and metastasis, RNA modification has also become a popular topic in cancer. Specifically, N4-acetylcytidine modification in other highly stable RNAs, such as circRNA, can be a possible mechanism of aberrant RNA modifications in HCC, which has rarely been studied in the current literature^59^. On the other hand, there might be a regulation network among immunodeficiency, a microbiome, and circRNA. Immunodeficiency can promote adaptive alterations of the host–gut microbiome and affect cancer development and progression^60^.

This study has some limitations. First, we did not examine hsa_circ_0005986 expression at a mechanistic level but rather evaluated the association between its expression and clinical endpoints. Further investigation of the mechanism of action of hsa_circ_0005986 is essential. Second, considering the retrospective nature of this study, there may have been some selection bias, considering that patients with missing medical records were not included. We excluded 39 patients who were either lost to follow-up or were previously treated for HCC, which reduced the total number of patients available for analysis. A larger number of patients are needed to validate the associations with hsa_circ_0005986 expression. It is necessary to improve the performance of hsa_circ_0005986 predicting prognoses, specifically for survival and progression. Third, percutaneous needle biopsy performed to obtain the specimens may not adequately reflect tumor heterogeneity. Some pathological features that affect the survival of patients with HCC cannot be assessed using needle biopsies (histological grade, microvascular invasion, and lymphatic invasion). This also reinforces the need to identify noninvasive biomarkers. Noninvasive diagnostic approaches such as serum or exosome collection are needed to validate whether hsa_circ_0005986 can be used as a prognostic biomarker in patients with HCC.

## Conclusions

In conclusion, our results showed the association between hsa_circ_0005986 expression and HCC proliferation and progression. Considering that hsa_circ_0005986 was shown to be a predictor of HCC progression and survival of patients with HCC, we believe that it has potential to become both a prognostic biomarker and a therapeutic target. However, additional studies are needed to clarify the mechanisms underlying the causal role of hsa_circ_0005986 in HCC progression under the Mendelian Randomization framework through integrating multi-omics datasets^61–63^. In addition, it is important to develop effective individualized therapeutic strategies to help improve the outcomes of patients with HCC.

## Supplementary Information

Supplementary Information.

## Data Availability

The datasets used or analysed during the current study are available from the corresponding authors on reasonable request.

## Abbreviations

CircRNA: Circular RNA
HCC: Hepatocellular carcinoma
OR: Odds ratio
CI: Confidence interval
HR: Hazard ratio
MiRNA: MicroRNA
MRE: MiRNA response element
EMT: Epithelial-mesenchymal transition
CT: Computed tomography
MRI: Magnetic resonance imaging
TNM: Tumor node metastasis
BCLC: Barcelona Clinic Liver Cancer
CTP: Child–Turcotte–Pugh
AFP: Alpha-fetoprotein
AUC: Area under the curve
ROC: Receiver operating characteristic
