# Supplementary figures and images for: Circular noncoding RNA hsa_circ_0005986 as a prognostic biomarker for hepatocellular carcinoma
Source: Sci Rep. 2021 Jul 22;11:14930. doi: 10.1038/s41598-021-94074-y (PMC8298461; doi:10.1038/s41598-021-94074-y)

**Supplementary Figure 1.** Primer design scheme for hsa\_circ\_0005986 detection

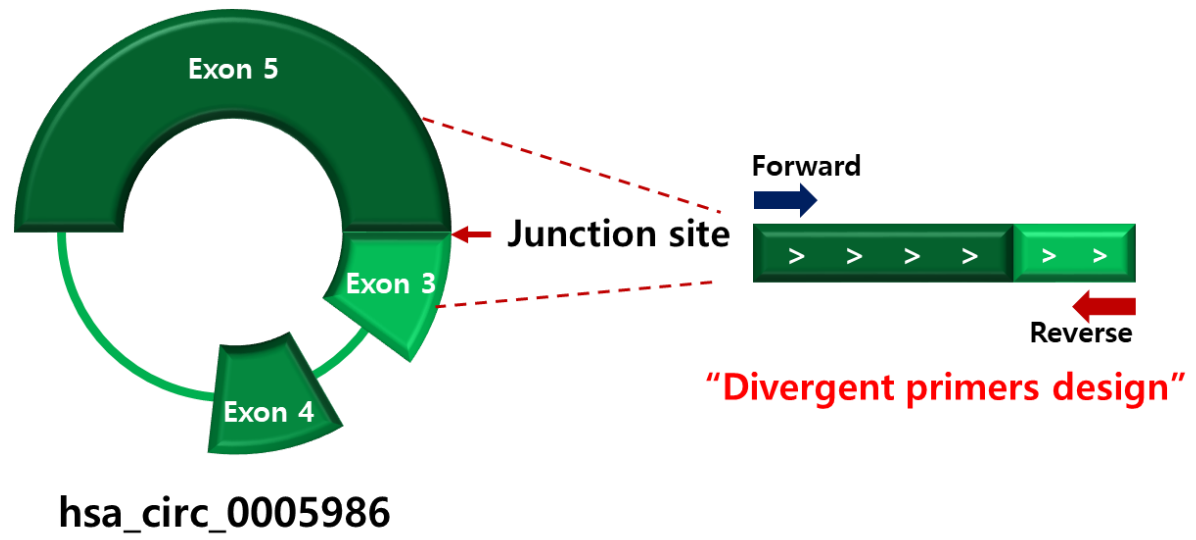

Supplement: Supplementary file 1 — Supplementary Information. [file 41598_2021_94074_MOESM1_ESM.pdf]
